# Biological Role of Anions (Sulfate, Nitrate , Oxalate and Acetate) on the Antibacterial Properties of Cobalt (II) and Nickel(II) Complexes With Pyrazinedicarboxaimide Derived, Furanyl and Thienyl Compounds

**DOI:** 10.1155/MBD.1999.95

**Published:** 1999

**Authors:** Zahid H. Chohan, M. Praveen

**Affiliations:** 1 Department of Chemistry Islamia University Bahawalpur Pakistan; 2 Department of Chemistry Washington University St. Louis 63130 USA

## Abstract

A number of biologically active complexes of cobalt(II) and nickel(II) with pyrazinedicarboxaimido derived thienyl and furanyl compounds having the same metal ion but different anions such as sulphate, nitrate, oxalate and acetate have been synthesized and characterized on the basis of their physical, spectral and analytical data. In order to evaluate the role of anions on their antibacterial properties, these ligands and their synthesized metal complexes with various anions have been screened against bacterial species *Escherichia coil*,*Pseudomonas aeruginosa* and *Staphylococcus aureus*. The title studies have proved a definitive role of anions in increasing the antibacterial properties.


### BIOLOGICAL ROLE OF ANIONS (SULFATE, NITRATE, OXALATE AND ACETATE) ON THE ANTIBACTERIAL PROPERTIES OF COBALT(II) AND NICKEL(II) COMPLEXES WITH PYRAZINEDICARBOXAIMIDE DERIVED, FURANYL AND THIENYL COMPOUNDS

Zahid H. Chohan*[1] and M. Praveen[2]

[1] Department of Chemistry, Islamia University, Bahawalpur, Pakistan
[2] Department of Chemistry, Washington University, St. Louis 63130, USA

**ABSTRACT:**
A number of biologically active complexes of cobalt(II) and nickel(II) with pyrazinedicarboxaimido derived thienyl and furanyl compounds having the same metal ion but different anions such as sulphate, nitrate, oxalate and acetate have been synthesized and characterized on the basis of their physical, spectral and analytical data. In order to evaluate the role of anions on their antibacterial properties, these ligands and their synthesized metal complexes with various anions have been screened against bacterial species *Escherichia coli, Pseudomonas aeruginosa* and *Staphylococcus aureus*. The title studies have proved a definitive role of anions in increasing the antibacterial properties.

### INTRODUCTION
Several reviews and studies in the literature[1-5] have discussed the role of metal ions in biological systems. There is no longer any controversy in researches[6-9] that certain types of cancers are viruses associated. The potentiality and affectivity of anticancer drugs have strongly shown their direct relationship[10-13] with the metal ions. Such a biological role of metal ions is, also, obvious and well known[14-16] in many antibacterial drugs. Our previous studies[17-20] have systematically elaborated this relationship of metal ions in subsequently increasing the activity of bactericidal compounds. This relationship of metal ions and the biochemical processes ought to gain awareness of those factors, which are responsible in enhancing the biological activity. In exploration of these factors, we, wish to report in the preceding paper the possible role of anions (sulfate, nitrate, oxalate and acetate) which stay as a counter part of the metal ion (cation) in the complex.

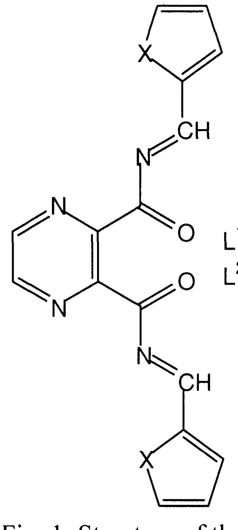

$L^1$: X = O
$L^2$: X = S

Fig. 1: Structure of the Ligand

### Experimental
### Materials and Methods
All chemicals and solvents used were of Analar grade. All the metals were used as their metal(II) chlorides. IR, $^1H$ NMR and $^{13}C$ NMR spectra were recorded on Philips Analytical PU 9800 FTIR and Brucker 250 MHz instruments. UV- Visible spectra were obtained on a Hitachi U-2000 double-beam spectrophotometer.

Conductance of the metal complexes was determined in DMF on a YSI-32 model conductometer. Magnetic measurements were done on solid complexes using the Gouy method. Melting points were recorded on a Gallenkamp apparatus and are uncorrected. The antibacterial studies were carried out with the help of the Department of Pathology, Quaid-e-Azam Medical College, Bahawalpur, Pakistan.

**Preparation of Ligands**

The ligands were prepared by adopting the same procedure as reported earlier[30] by us. The structural determination of these ligands was done with the help of their IR, $^1$H-NMR, $^{13}$C-NMR and microanalytical data.

**Preparation of Metal Complexes**

To a hot n-butanol solution (30 mL) of the ligand (0.02 mol) was added an ethanolic solution (25 mL) of the respective metal(II) salt (0.01 mol). The mixture was refluxed for 2 h. The resulting mixture was cooled, filtered and reduced to half of its volume (20 mL). The concentrated solution so obtained was left overnight at room temperature which resulted in the formation of a solid product. The product thus formed was filtered, washed with n-butanol (2x10 mL), then with ethanol (2x10 mL) followed by ether (2x20 mL) and dried. Crystallization in hot aqueous n-butanol (50 %) gave **1** (68 %), **2** (65 %), **3** (65 %), **4** (67 %), **5** (65 %), **6** (69 %), **7** (65 %), **8** (60), **9** (58 %), **10** (63 %), **11** (65 %), **12** (65 %), **13** (67 %), **14** (68 %), **15** (65 %) and **16** (66 %).

**Antibacterial Studies**

**Preparation of Discs.**

The ligand/complex (30 µg) in DMF (0.01mL) was applied on a paper disc, [prepared from blotting paper (3 mm diameter)] with the help of a micropipette. The discs were left in an incubator for 48 h at 37° C and then applied on the bacteria grown agar plates.

**Preparation of Agar Plates.**

Minimal agar was used for the growth of specific bacterial species. For the preparation of agar plates for *Escherichia coli*, MacConkey agar (50 g), obtained from Merck Chemical Company, was suspended in freshly distilled water (1 L). It was allowed to soak for 15 minutes and then boiled on a water bath until the agar was completely dissolved. The mixture was autoclaved for 15 minutes at 120° C and then poured into previously washed and sterilized Petri dishes and stored at 40° C for inoculation.

**Procedure of Inoculation.**

Inoculation was done with the help of a platinum wire loop which was made red hot in a flame, cooled and then use for the application of bacterial strains.

**Application of Discs.**

A sterilized forceps was used for the application of paper disc on the already inoculated agar plates. When the discs were applied, they were incubated at 37° C for 24 h. The zone of inhibition was then measured (in diameter) around the disc.

### RESULTS AND DISCUSSION

All the metal complexes were synthesized by a stoichiometric reaction of metal(II) salt having different anions and the respective ligands in a molar ratio 1 : 2 = M : L (metal : ligand). All complexes are air and moisture stable solids, soluble in DMF, DMSO and water and partially soluble in ethanol, acetone and benzene. The conductivity measurements (20-28 $ohm^{-1}$ $cm^2$ $mol^{-1}$) of these complexes in DMF indicated[21,22] them to be non-electrolytes.

**Infrared Spectra**

The bonding of the ligand to the metal ion was investigated by mainly comparing the infrared spectra of the free ligands with the spectra of their metal complexes. The infrared spectra show a positive shift of bands at 1510 $cm^{-1}$ due to skeletal modes of the pyrazine ring, indicating[23] co-ordination through pyrazine ring nitrogen. Also, an insignificant shift of ν(C=O) at 1670 $cm^{-1}$ indicated that it is not co-ordinated to the metal atom. The spectra of all complexes (Table 2) also indicated that a band at 1640 $cm^{-1}$ due to azomethine ν(C=N) linkage was shifted towards lower frequency by 5-10 $cm^{-1}$, respectively, suggesting that the ligands are involved in co-ordination to the metal atom via the azomethine nitrogen. The new bands appearing in the spectra of metal complexes and not observed in the spectra of the ligands within 442-455 $cm^{-1}$ and 375-386 $cm^{-1}$ assigned to M-O and M-S modes, respectively, gave a definitive clue[24] that the heteroatoms X (Fig 1) are also involved in the co-ordination.

**Magnetic Moment**

The room temperature magnetic susceptibility measurements (Table 1) for the solid complexes lie within the range expected for their octahedral geometry. Three unpaired electrons per Co(II) ion ($\mu_{eff}$=4.35-4.87 B.M) and two unpaired electrons per Ni(II) ion ($\mu_{eff}$=2.85-3.24 B.M) suggested[25,26] octahedral geometry for Co(II) and Ni(II) complexes.

Table 1 Physical And Analytical Data of Metal Chelates

| No | Metal chelate/ Mol. Formula | M.P(°C) (decom) | B.M ($\mu_{eff}$) | Calc (Found)% C H N |
|---|---|---|---|---|
| 1 | $[Co(L^1)_2(SO_4)_2]$ $C_{32}H_{20}CoN_4O_{12}S_2$ [895.37] | 209-211 | 4.68 | 42.9 2.2 6.2 (43.3) (2.5) (6.6) |
| 2 | $[Co(L^1)_2(NO_3)]$ $C_{32}H_{20}CoN_5O_7$ [765.25] | 217-219 | 4.45 | 50.2 2.6 9.1 (49.9) (2.6) (9.4) |
| 3 | $[Co(L^1)_2(C_2O_4)_2]$ $C_{36}H_{20}CoN_4O_{12}$ [879.25] | 201-203 | 4.35 | 49.2 2.3 6.4 (50.1) (2.9) (6.8) |
| 4 | $[Co(L^1)_2(CH_3CO_2)]$ $C_{34}H_{23}N_4O_6$ [762.25] | 228-230 | 4.87 | 53.6 3.0 7.3 (53.2) (2.8) (7.6) |
| 5 | $[Co(L^2)_2(SO_4)_2]$ $C_{32}H_{20}CoN_4O_{10}S_2$ [959.61] | 233-235 | 4.64 | 40.0 2.1 5.8 (39.7) (2.2) (5.5) |
| 6 | $[Co(L^2)_2(NO_3)]$ $C_{32}H_{20}CoN_5O_5S$ [825.49] | 210-212 | 4.82 | 46.3 2.4 8.4 (46.2) (2.3) (8.9) |
| 7 | $[Co(L^2)_2(C_2O_4)_2]$ $C_{36}H_{20}CoN_4O_{10}S$ [943.49] | 225-227 | 4.55 | 45.8 2.1 5.9 (46.1) (2.4) (6.0) |
| 8 | $[Co(L^2)_2(CH_3CO_2)]$ $C_{34}H_{23}CoN_4O_4S$ [826.49] | 222-224 | 4.85 | 49.4 2.8 6.8 (49.2) (2.5) (6.7) |
| 9 | $[Ni(L^1)_2(SO_4)_2]$ $C_{32}H_{20}NiN_4O_{12}S_2$ [895.13] | 228-230 | 2.85 | 42.9 2.2 6.3 (42.7) (2.2) (6.7) |
| 10 | $[Ni(L^1)_2(NO_3)]$ $C_{32}H_{20}NiN_5O_7$ [765.01] | 218-220 | 2.91 | 50.2 2.6 9.2 (50.5) (2.8) (9.4) |
| 11 | $[Ni(L^1)_2(C_2O_4)_2]$ $C_{36}H_{20}NiN_4O_{12}$ [879.01] | 202-204 | 3.24 | 49.2 2.3 6.4 (49.4) (2.5) (6.1) |
| 12 | $[Ni(L^1)_2(CH_3CO_2)]$ $C_{34}H_{23}NiN_4O_6$ [762.01] | 210-212 | 3.22 | 53.6 3.0 7.3 (53.4) (3.1) (7.6) |
| 13 | $[Ni(L^2)_2(SO_4)_2]$ $C_{32}H_{20}NiN_4O_{10}S_2$ [959.37] | 225-227 | 2.95 | 40.1 2.1 5.8 (39.8) (2.3) (5.5) |
| 14 | $[Ni(L^2)_2(NO_3)]$ $C_{32}H_{20}NiN_5O_5S$ [829.25] | 213-215 | 3.12 | 46.3 2.4 8.4 (46.8) (2.5) (8.2) |
| 15 | $[Ni(L^2)_2(C_2O_4)_2]$ $C_{36}H_{20}NiN_4O_{10}S$ [943.25] | 206-208 | 2.88 | 45.8 2.1 5.9 (45.6) (2.0) (6.2) |
| 16 | $[Ni(L^2)_2(CH_3CO_2)]$ $C_{32}H_{20}NiN_4O_{12}S_2$ [826.25] | 211-213 | 2.97 | 46.5 2.4 6.7 (46.2) (2.3) (6.7) |

**Electronic Spectra**

Electronic spectra of the metal complexes are recorded in Table 2. The spectra of the cobalt chelates show three bands observed at 8725-9555 $cm^{-1}$, 17365-18285 $cm^{-1}$ and 29515-31255 $cm^{-1}$ which may be assigned[27] to $^4T_1g \rightarrow {}^4T_2g$ (F), $^4T_1g \rightarrow {}^4A_2g$ (F) and $^4T_1g \rightarrow {}^4T_1g$ (P) transitions, respectively, and are suggestive[28] of octahedral geometry around the cobalt ion. Three bands observed at 9315-10312 cm$^{-1}$, 15870-17125 cm$^{-1}$ and 26355-27550 cm$^{-1}$ in the spectra of the nickel(II) chelates are due to spin-allowed transitions from $^3A_2g \rightarrow {}^3T_2g$ (F), $^3A_2g \rightarrow {}^3A_1g$ (F) and $^3A_2g \rightarrow {}^3T_1g$ (P), transitions, respectively, in an octahedral environment[29].

Based on the above evidences, it is proposed that cobalt(II) and nickel(II) complexes have octahedral geometry in which the ligands behave as tridentate and accommodate themselves in such a way that a stable chelate ring is formed around the metal atom thus attaining a stable configuration.

Table 2 Spectral Data of Metal Chelates

| No | IR (cm$^{-1}$) | $\lambda_{max}$ (cm$^{-1}$) |
|---|---|---|
| 1 | 1670 (C=O), 1630 (C=N), 1515 (pyrazine),445 (M-O) | 29535, 18285, 8765 |
| 2 | 1670 (C=O), 1635 (C=N), 1530 (pyrazine), 442 (M-O) | 30865, 17365, 9555 |
| 3 | 1670 (C=O), 1635 (C=N), 1533 (pyrazine) 442 (M-O) | 30260, 18150, 8795 |
| 4 | 1670 (C=O), 1632 (C=N), 1525 (pyrazine), 445 (M-O) | 31255, 18185, 9390 |
| 5 | 1670 (C=O), 1635 (C=N), 1530 (pyrazine), 375 (M-S) | 29715, 17422, 9550 |
| 6 | 1670 (C=O), 1635 (C=N), 1532 (pyrazine), 380 (M-S) | 30525, 18257, 9255 |
| 7 | 1670 (C=O), 1630 (C=N), 1535 (pyrazine) 385 (M-S) | 30115, 17720, 9315 |
| 8 | 1670 (C=O), 1635 (C=N), 1535 (pyrazine), 382 (M-S) | 29575, 17522, 8905 |
| 9 | 1670 (C=O), 1635 (C=N), 1537 (pyrazine), 445 (M-O) | 26355, 15870, 9315 |
| 10 | 1670 (C=O), 1635 (C=N), 1535 (pyrazine), 445 (M-O) | 27550, 17125, 10312 |
| 11 | 1670 (C=O), 1630 (C=N), 1535 (pyrazine), 445 (M-O) | 26565, 16288, 9870 |
| 12 | 1670 (C=O), 1635 (C=N), 1535 (pyrazine), 445 (M-O) | 27235, 16542, 9755 |
| 13 | 1670 (C=O), 1635 (C=N), 1535 (pyrazine), 375 (M-S) | 26910, 16111, 9785 |
| 14 | 1670 (C=O), 1630 (C=N), 1535 (pyrazine), 380 (M-O) | 27222, 17085, 9412 |
| 15 | 1670 (C=O), 1635 (C=N), 1535 (pyrazine), 385 (M-O) | 26721, 16295, 10188 |
| 16 | 1670 (C=O), 1630 (C=N), 1535 (pyrazine), 385 (M-O) | 27445, 15770, 9437 |

Table 3 Antibacterial Activity Data

| Ligand/Chelate | Microbial Species | | |
|---|---|---|---|
| | a | b | c |
| $L^1$ | ++ | + | + |
| $L^2$ | + | ++ | + |
| 1 | ++++ | ++ | +++ |
| 2 | +++ | +++ | ++ |
| 3 | +++ | +++ | +++ |
| 4 | ++ | ++++ | +++ |
| 5 | +++ | ++++ | +++ |
| 6 | +++ | +++ | ++ |
| 7 | +++ | +++ | +++ |
| 8 | +++ | +++ | +++ |
| 9 | ++++ | ++++ | +++ |
| 10 | ++ | ++ | + |
| 11 | +++ | +++ | ++ |
| 12 | +++ | ++ | +++ |
| 13 | +++ | ++ | +++ |
| 14 | +++ | +++ | +++ |
| 15 | ++ | ++ | ++++ |
| 16 | +++ | +++ | ++ |

a=*Escherichia coli*, b=*Pseudomonas aeruginosa*, c=*Staphylococcus aureus*
Inhibition zone diameter mm (% inhibition): +, 6-10 (27-45 %); ++, 10-14 (45-64 %);
+++, 14-18 (64-82 %); ++++, 18-22 (82-100 %). Percent inhibition values are relative to inhibition zone (22 mm) of the most active compound with 100 % inhibition.

**Antibacterial Studies**

The title ligands in comparison to their metal complexes were screened against bacterial species *Escherichia coli, Pseudomonas aeruginosa* and *Staphylococcus aureus* in order to determine their antibacterial properties. The antibacterial activity was tested at a concentration 30μg/0/01 mL in DMF using paper disc diffusion method. The results of these studies reproduced in Table 3 showed that ligands and all their metal complexes are biologically active against one or more bacterial species and the metal complexes having different anions have been shown to be more antibacterial than the simple uncomplexed parent ligands.

These results clearly indicated that when the same metal chelate having different anions was individually screened, the degree of bactericidal activity varied. For example, the Co(II) complex having nitrate as anion was more bactericidal than the Co(II) complex with sulphate, oxalate or acetate anions. Similarly, the Co(II) complex of oxalate anion was more antibacterial active than the complex with acetate, chloride or sulphate anions. The same results were found for Ni(II) complexes. From the obtained data, it was generally observed that the order of potency in comparison to the metal complexes having chloride anions evaluated and reported earlier[30] and to the results of the present studies for the anions sulphate, nitrate, oxalate and acetate against the tested bacterial species was found to follow the order as:

$$NO_3 > C_2O_4 > CH_3CO_2 > Cl > SO_4.$$

On the basis of these results, we strongly claim that different anions do effect the biological behavior of the metal chelates. However, at this stage, we are not aware of the actual mechanism. It is however, suspected that factors such as solubility, conductivity, dipole moment and cell permeability mechanism (influenced by the presence of anions) may be the possible reasons for effecting the activity.

**Acknowledgement**

We are grateful to the Department of Pathology, Qaid-e-Azam Medical College, Bahawalpur, for the help in undertaking the antibacterial studies.

**References**

1. .W. N. Lipscomb, *Chem. Soc. Rev.*, **1**, 319 (1972).
2. B. Rosenberg, *Mot. Rev.*, **15**, 42 (1971).
3. T. A. Connors, *Recent Results Cancer Res.*, **48**, 113 (1974).
4. A. Lodzinska, F. Golinska, F. Rozploch and A. Jesmanowier, *Pol. J. Chem.*, **60**, 389 (1986).
5. S. E. Livingstone and J. D. Nolan, *J. Inorg. Chem.*, 7, 1447 (1968).
6. B. Rosenberg, *Plat. Met. Rev.*, **15**, 42 (1971).
7. D. R. Williams, "*The Metals of Life*", Van Nostrand, London (1971).
8. R. J. P. Williams, *"Bioinorganic Chemistry"*, Adv. In Chemistry. No 100, Amer. Chem. Soc, Washington (1971).
9. R. J. P. Williams, *Quart. Rev.*, **72**, 203 (1972).
10. D. R. Williams, *Chem. Rev.*, **17**, 113 (1972).
11. G. M. Timmis and D. C. Williams, "*Chemotherapy of Cancer*", Butterworths, London (1967).
12. M. J. Seven and L. A. Johnson, "*Metal Binding in Medicine*", Lippincott Co, Philadelphia, Pa (1960).
13. B. Rosenberg and L. V. Camp, *Cancer. Res.*, **30**, 1799 (1970).
14. V. K. Revankar and V. B. Mahal, *Ind. J. Chem.*, **28A**, 683 (1989).
15. R. P. Gupta, B. N. Yadav, O. P. Tiwari and A. K. Sirivastava, *Inorg. Chim. Acta.*, **32**, L95 (1979).
16. M. Melnik, M. Auderova and M. Holko, *Inorg. Chim. Acta.*, **67**, 117 (1982).
17. Z. H. Chohan, M. Praveen and A. Ghaffar, *Metal-Based Drugs.*, **4**, 267 (1997).
18. Z. H. Chohan and H. Pervez, *Synth. React. Inorg. Met-Org. Chem.*, **23**, 1061 (1993).
19. Z. H. Chohan and S. K. A. Sherazi, *Metal-Based Drugs.*, **4**, 65 (1997).
20. Z. H. Chohan and A. Rauf, *J. Inorg. Biochem.*, **46**, 41 (1992).
21. Z. H. Chohan and S. K. A. Sherazi, *Metal-Based Drugs.*, **4**, 69 (1997).
22. W. J. Geary, *Coord. Chem. Rev.*, 7, 81 (1971).
23. A. M. Shallary, M. M. Moustafa and M. M. Bekheit, *J. Inorg. Nucl. Chem.*, **41**, 267

**Received: March 8, 1999 - Accepted: March 11, 1999 - Received in revised camera-ready format: March 13, 1999**

